# Comparative Study of The Effect of LPS on The Function of BALB/c and C57BL/6 Peritoneal Macrophages

**Published:** 2013-05-05

**Authors:** Sara Soudi, Ahmad Zavaran-Hosseini, Zuhair Muhammad Hassan, Masoud Soleimani, Fatemeh Jamshidi Adegani, Seyed Mahmoud Hashemi

**Affiliations:** 1 Department of Immunology, Faculty of Medical Sciences, Tarbiat Modares University, Tehran, Iran; 2 Department of Hematology, Faculty of Medical Sciences, Tarbiat Modares University, Tehran, Iran; 3 Department of Stem Cell Biology, Stem Cell Technology Research Center, Tehran, Iran

**Keywords:** Macrophage, Thioglycolate, LPS, BALB/c, C57BL/6

## Abstract

**Objective:**

Macrophages influence their environment and surrounding immune cells as soon as stimulators affect them. Different sources of macrophages induce different reactions in their neighboring immune cells,which result in non-uniform immunologic outcomes. In this experimental research, we compare the behavior of peritoneal macrophages to lipopolysaccharide (LPS) stimulation from BALB/cmice as an indicator of a type 2 immune response and from C57BL/6 mice as an indicator of a type 1 immune response.

**Materials and Methods:**

In this experimental study, peritoneal macrophages prepared from thioglycolate stimulated BALB/c and C57BL/6 micewere treated with 1µg/ml LPS. At different time points after LPS treatment, nitric oxide (NO), interferon gamma (IFN-λ), interleukin 4 (IL-4),transforming growth factor β_1_(TGF-β_1_), interleukin 17 (IL-17), and interleukin 10(IL-10) production were measured in the supernatants of all macrophage cultures. Indoleamine 2, 3 dioxygenase (IDO) and phagocytic activitywere analyzed in the different experimental groups. The supernatant effects of LPS-treated macrophages on splenocyte proliferation was assessed by the colorimetric method using a 3-(4,5-Dimethylthiazol- 2-yl)-2, 5-diphenyltetrazolium bromide (MTT) reagent.

**Results:**

According to cytokine analysis, different mouse strains show different cytokine patterns in response to LPS. C57BL/6 macrophages produced more IL-17, IL-10, and IFN-λ, while BALB/c macrophages produced more TGF-β_1_ and IL-4. There was no significant difference in IDO activity between strains (p≤0.05). BALB/c mice produced more NO inthe first 24 hours after LPS treatment,but C57BL/6 produced more NO at 72 hours post-LPS treatment. Macrophages from both strains hada suppressor effect on splenocyte proliferation, but this effect was stronger in BALB/c mice.

**Conclusion:**

The results show that macrophages from different genetic backgrounds respond differently to the same stimulus in aspects of type, intensity, and time of response. The consideration of these aspects will enableresearchers to use correct treatment programs for immune-regulation or immunotherapy.

## Introduction

Macrophages are major responders of innate immunity by expression of innate immunity receptors and their ability to sense host and pathogenderived factors (1, 2). Macrophages are phagocytic cells that act both as a host for immune evasion of intra-cellular pathogens and as promoters to prime a specific immune response by antigen presentation and secretion of pro-inflammatory cytokines and chemokines (3). Experimental studies show that the early immune responses appearing after a pathogenic challenge determine the polarization of T cell responses and disease outcome. Different early immune responses mediated with the help of macrophages assist with induction of cellular immunity andthe control of immune homeostasis (4). In every immune stimulation or infection, a heterogeneous population of macrophages will activate and produce pro-inflammatory to anti-inflammatory responses, depending on the nature of the stimulators and the induced signaling pathways. Evidence shows that macrophages from Th1 strains (C57BL/6) or Th2 strains (BALB/c) of mice are activated in different ways in response to the same stimuli,guiding the immune responses in opposing directions (4, 5). According to this functional diversity, macrophages can be divided into distinct groups, M-1 and M-2 that influence the formation of Th1/Th2 and other inflammatory responses (4, 5). Because of the variety in macrophage function of different strains, and the key role of macrophages in infections and inflammatory disease, targeted studies should be designed to clarify the role of macrophages under different conditions. The obtained results will create a helpful map for better manipulation of macrophages by using suitable treatments or stimuli.

Much of the current knowledge on the function of macrophages in response to immunomodulatorshas been derived from the analysis of the RAW 264.7 cell line. This cell linehas been established from the ascites of tumor-induced BALB/c mice (6, 7). However these results are not extendable tothose macrophages that havebeen separated from distinct anatomical parts of the body,and therefore have different characteristics. Splenic, alveolar, or peritoneal macrophages are examples of distinct anatomic macrophage sources (8, 9). Researchers have shown that large peritoneal macrophages (LPMs) and small peritoneal macrophages (SPMs) are two developmentally and functionally distinct macrophage subsets that respond differently to typical stimuli, such as lipopolysaccharides (LPS) (9). LPSis a pathogen-specific molecular motif that, after interaction with toll-like receptor-4 (TLR-4) on macrophages, induces macrophage migration to the infected site through actin reassembly (10). Following genetic reprogramming in activated macrophages the expression of TLR-4 mediated signaling genes isup-regulated that terminated to the activation ofnuclear factor κB (NFκB),mitogen activated protein kinases (MAP kinases), and finally the expression of pro-inflammatory cytokine genes (1, 6, 11).

The goal of our study was to compare peritoneal macrophage responsesto LPS stimulation from two different genetic backgrounds over a determined period of time.Thioglycolate stimulation was used to induce inflammation and migration of blood monocytes to the peritoneal fluid (9). At different times after LPS treatment,we measured phagocytic activity, cytokine pattern, IDO activity, and nitric oxide (NO) production of the macrophages.

## Materials and Methods

### Mice

Six, eight-week-oldfemale BALB/c and C57BL/6 mice were purchased from Pasteur Institute of Iran. Mice wereused for splenocyte and peritoneal macrophage preparation. All animal experiments followed the guidelines of the Laboratory Animal Ethical Commission of Tarbiat Modares University.

### Macrophage preparation

Peritoneal macrophages from BALB/c and C57BL/6 mice were obtainedby the peritoneal lavage technique.Four days after peritoneal injection of 4% w/v thioglycolate medium (2 ml), mice were ethically sacrificed by CO_2_ inhalation.A total of 8 ml of cold RPMI-1640, supplemented with 1% penicillin-streptomycin (Gibco, Grand Island, NY, USA)was injected into the peritoneal cavityin the lower abdominal area near the fat region. After needle withdrawal, mice were held by theirtails and swishedaround to wash the peritoneal cavity. The needle was inserted into the upper part of the abdomen and peritoneal fluids were collected. The resultant cell suspension was centrifuged at 350×g for 5 minutes and adjusted to1×10^6^ cells/ ml in RPMI-1640 (Sigma-Aldrich, St. Louis, MO, USA) that contained 10% fetal bovine serum (FBS), 2 mM of 1% L-glutamine, 10 mM of 1% HEPES, and 1% penicillin-streptomycin (Gibco, Grand Island, NY, USA). Aliquots of each sample (200 µl) were cultured in 4-well plates and incubated at 37˚C and 5% CO_2_. After 6 hours, the cells were washed to separate out any nonadhesive cells.Macrophage purity was determined by flow-cytometry analysis for CD14^+^ and CD11b^+^ markers.

### Lipopolysaccharide (LPS) treatment

For *in vitro* stimulation,we added LPS to a final concentration of 1 µg/ml to the macrophage cultures. This step was performedin triplicate. We assessed immunological parameters of themacrophage culture supernatants at 0 (just prior to LPS addition), and 3, 24, 48, and 72 hourspost- LPS treatment. The immunomodulatory effect of the immune responses on splenocytes was measured by culturing thesplenocytes in the supernatant of LPS-treated macrophages. The lack of LPS in the reagents and cell culture media wasconfirmed by the limulus amebocyte lysate (LAL) test.

### Cytokine measurement

Supernatants of the LPS-stimulated macrophages were collected at 0 (pre-treatment),and 3, 24, 48, and 72 hours post-LPS treatment. Supernatants from different experiments were stored at -20˚C. The presence of interferon gamma (IFN-λ), interleukin 4 (IL-4), transforming growth factor β_1_ (TGF-β_1_), interleukin 17 (IL-17), and interleukin 10 (IL-10) cytokines were assessed using enzyme-linked immunosorbent assay (ELISA) kits from eBioscience, following the manufacturer’s instructions.Each sample was dispensed in triplicate. The optical density of each well was determined at 450 nm. We used Microsoft Excel to draw standard curves and ELISA results.

### Indoleamine 2, 3 dioxygenase (IDO) activity assay

Indoleamine 2, 3 dioxygenase (IDO) activity was determined bythe colorimetric assay that measured the amount of kynurenine, the first stable catabolite downstream of the enzyme’s activity. Briefly, 100 µl of 30% trichloroacetic acid was added to 200 µl culture supernatants of the control and test groups. After centrifugation at 8000×g, 75 µl of the supernatant from each sample was added to an equal volume of Ehrlich reagent (100 mg P-dimethylbenzaldehyde, 5 mL glacial acetic acid) in microliter 96-well plates, in triplicate. Optical density was measured at 492 nm. We calculated the amount of kynurenine according to the standard curve of defined kynurenine concentration (0-100 µM).

### Nitric oxide (NO) measurement

All supernatants from LPS-treated macrophages were collected and stored at-20˚C. (This has already been mentioned. One should be deleted.) Nitrite was measured by addition of 100µl of Griess reagent (1% sulphanilamide and 0.1% naphthylenediamine in 5% phosphoric acid; Sigma) to 100 µl of the supernatants. Optical density was read at 540 nm and NO concentration was determined by using the standard concentrations of sodium nitrite (0-100 µM).

### Splenocyte proliferation assay

BALB/c×C57BL/6 hybrid mice (4) spleens were removed and homogenized in 2 ml cell culture media. After centrifugation, erythrocytes were lysed bylysis buffer that consisted of NH_4_Cl (0.15 M), KHCO3 (1.0 mM), and Na2EDTA (0.1 mM). The splenocytes were washed three times in media and cultured in RPMI-1640 (Sigma-Aldrich, St. Louis, MO, USA) that contained 5% FBS, 1% Lglutamine (2 mM), 1% HEPES (10 mM), and 1% penicillin-streptomycin (Gibco, Grand Island, NY, USA). Splenocytes were counted and adjusted to 5×10^5^ cells/ml. A total of 50µl of the cell suspension was co-cultured with 150 µl of the 0 (pretreatment), and 3, 24, 48, and 72 hour LPS-treated macrophage supernatants. Each sample was tested in triplicate. After incubation for 7 hours at 37˚C and 5%CO_2_, 10 µl 3- (4, 5-Dimethylthiazol- 2-yl)-2, 5-diphenyltetrazolium bromide (MTT; Sigma-Aldrich, St. Louis, MO, USA) was added to each well and the plates were incubated for an additional 6 hours (12). Formazan crystals were dissolved in 200 µl of dimethyl sulfoxide (DMSO; Sigma-Aldrich, St. Louis, MO, USA). Optical density was read at 540 nm on the plate reader usinga reference wavelength of 630 nm. Cell count was determined according to standard curve.

### Phagocytosis assay

Peritoneal macrophages from BALB/c and C57BL/6 mice were seeded at a concentration of 10×10^4^) cells/well in a 4-well plate. After 0 (pretreatment), and 3, 24, 48, and 72 hours of LPS treatment, cells were washed and 4', 6-diamidino- 2-phenylindole (DAPI)-stained Saccharomyces cerevisiae were added at a ratio of 5:1 cells to each well. Cells were incubated for 1hour at 37˚C and 5% CO_2_ , then washed carefully to remove all yeast particles. Control and test samples were examined under an inverted fluorescence microscope. At least 200 cells were counted and the percent of infected cells and phagocytic index was reported.

### Statistical analysis

Data analysis was performed with SPSS Statistical Package (SPSS Inc., Chicago, IL, USA) version 11.0. Differences between means were assessed for statistical differences using Mann-Whitney non-parametric assays and one-way analysis of variance (ANOVA). P<0.05 was statistically significant. The results are shown asmean ± standard deviation (SD).

## Results

### Flowcytometry

**Fig 1:**
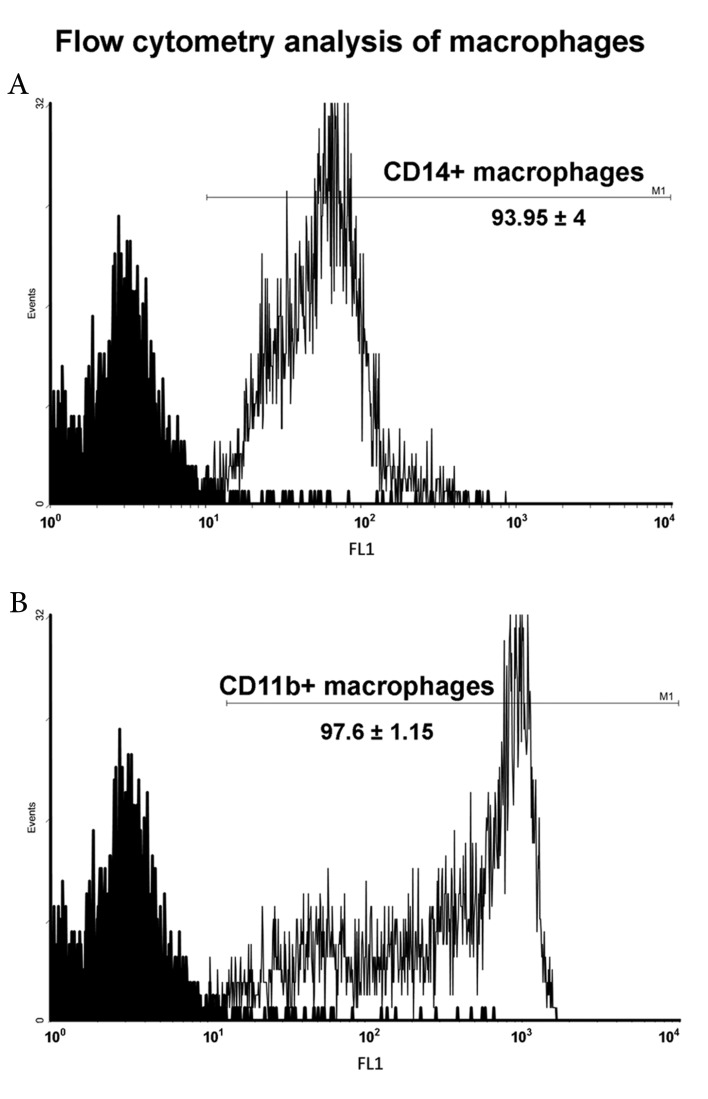
Flow cytometry analysis of peritoneal macrophages for CD11b and CD14 markers. Data shows the average number of positive cellsforfivemice per experimental group ± standard deviation.

We used various samples of prepared peritoneal macrophages from BALB/c and C57BL/6 mice for the flowcytometry measurement of macrophage specific cellsurface markers, CD14 and CD11b. As indicated in figure 1, the mean percentage of CD14 positive cells was 93.95 ± 4, and the mean percentage of CD11b positive cells was 97.6 ± 1.15.

### Cytokine assay

**Fig 2:**
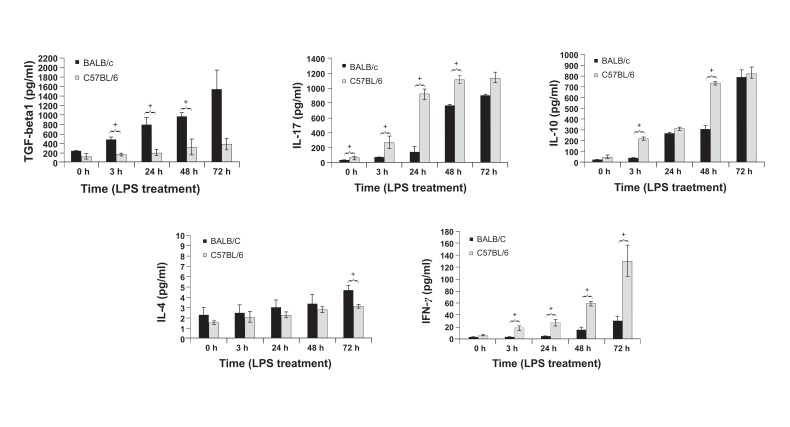
TGF-β_1_, IL-17, IL-10, 1L-4, and IFN-λ production by LPS-stimulated BALB/c and C57BL/6 macrophages at 0 (pretreatment), and 3, 24, 48, and 72 hours post-LPS treatment. The results represent the mean cytokine levels (pg/ml) forfive mice per experimentalgroup in triplicate ± tandard deviation. The single star on top of the bars shows the significant difference between BALB/c and C57BL/6 groups.

To determine the nature of the immune responses of macrophages from BALB/c and C57BL/6 mice to LPS stimulation, we measured the amount of IFN-λ, IL-4, TGF-β_1_, IL-17, and IL-10 produced in the supernatant at 0 (pre-LPS treatment), and 3, 24, 48, and 72 hours post- LPS treatment. As shown in figure 2, TGF-β_1_ production increased significantly (p≤0.05) in BALB/c macrophages compared to C57BL/6 macrophages in response to LPS. Cytokine analysis showed that IL-17 production was induced in both mice groups after LPS stimulation, but the production level of C57BL/6 macrophages was significantly higher than the production level of BALB/c macrophages at 0 (pre-stimulation), and 3, 24, and 48 hours poststimulation. A significant increase in IL-17 production occurred at 3 hours post-LPS treatment in C57BL/6 macrophages, but it occurredlater in BALB/c macrophages (48 hours). According to figure 2, IL-10 production was induced in both groups of mice; however, it was significantly higher in C57BL/6 macrophages compared to BALB/c macrophages at 3 and 48 hours post-stimulation. A significant increase in IL-10 production occurred at 3 hours post-LPS treatment in C57BL/6 macrophages; however, this occurred later in BALB/c macrophages (24 hours). A small increase (about 2 pg) in IL-4 production was detected after LPS stimulation,which was significantly higher in BALB/c macrophages compared to C57BL/6 macrophages at 72 hours post-treatment. IFN-λ production increased significantly in C57BL/6 macrophages compared to BALB/c macrophages at 3, 24, 48 and 72 hours post-LPS treatment.

### Measurement of indoleamine 2, 3 dioxygenase activity

**Fig 3:**
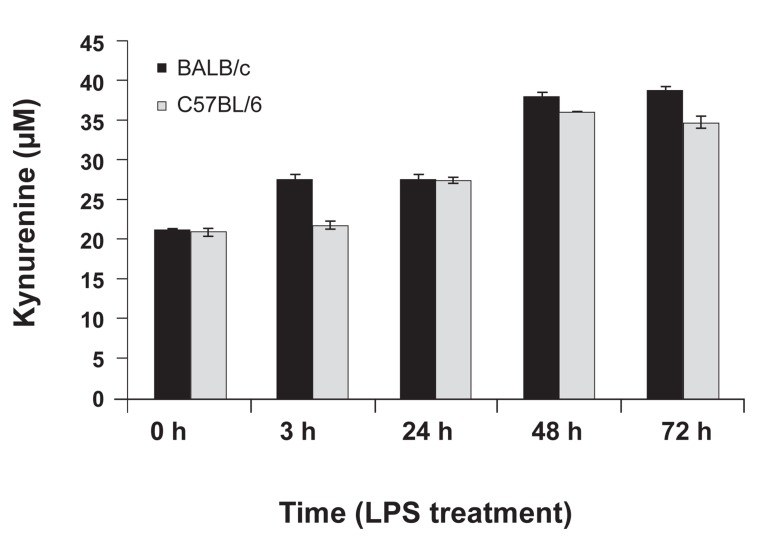
Kynurenine production by BALB/c and C57BL/6 peritoneal macrophages at 0 (pre-treatment), and 3, 24, 48, and 72 hours post-LPS treatment. Data shows the mean kynurenine level (µM) for five mice per experimentalgroup in triplicate ± standard deviation.

IDO activity was assessed by kynurenine measurement in the supernatant of LPS-stimulated macrophages. According to figure 3, IDO activity increased in both mice groups after LPS treatment, but this increase was not statisticallysignificant. There was no difference between BALB/c and C57BL/6 macrophages in kynurenine production.

### Nitric oxide measurement

**Fig 4:**
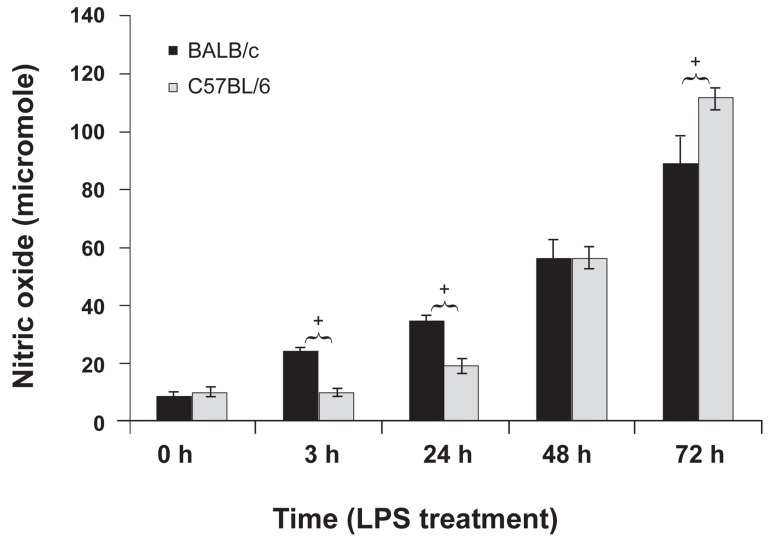
Nitric oxide (NO) production by BALB/c and C57BL/6 peritoneal macrophages at 0 (pre-treatment), and 3, 24, 48 and 72 hours post-LPS treatment. Data shows the mean NO level (µM) for five mice per experimental group in triplicate ± standard deviation. The single star on top of the bars showsthe significant difference between BALB/c and C57BL/6 groups.

NO production was measured in the supernatant of BALB/c and C57BL/6 macrophages at different time points after LPS treatment. NO production increased significantly in both groups after LPS stimulation (Fig 4). The increasing pattern of NO production at different time points differed betweenBALB/c and C57BL/6 macrophages. At 3 and 24 hours after LPS stimulation, BALB/c macrophages produced significantly higher amounts of NO than C57BL/6 macrophages. However,at 72 hours after LPS stimulation C57BL/6 macrophages produced significantly higher amounts of NO than BALB/c macrophages.

### Macrophage supernatant effect on splenocyte proliferation

**Fig 5:**
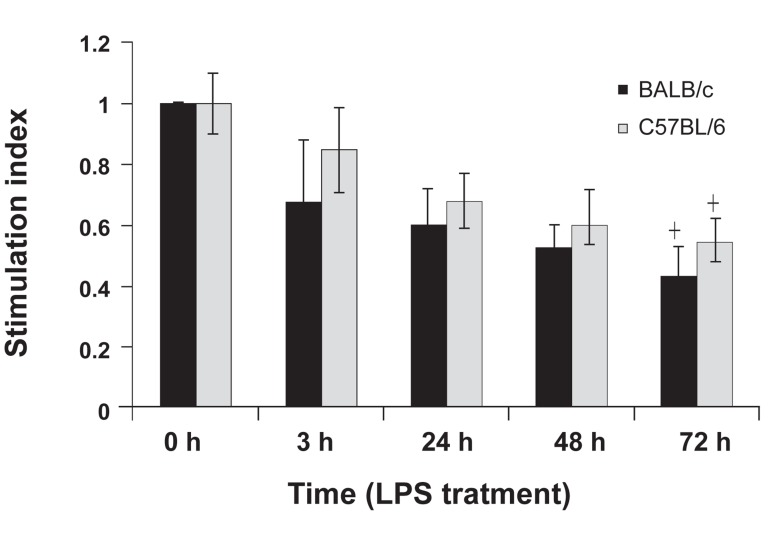
Lymphoproliferative responses of F1 hybrid mice (BALB/c x C57BL/6) spleen cells to the supernatants of BALB/c and C57BL/6 peritoneal macrophages at 0 (pretreatment), and 3, 24, 48, and 72 hours post-LPS treatment. The results represent the average of SI for five mice per ex perimental group in triplicate ± standard deviation.

An MTT assay was used to determine the effects of the supernatant from LPS-treated BALB/c and C57BL/6 macrophages on splenocyte proliferation. According to the stimulation index in Fig5, proliferation of splenocytes was inhibited in the presence of the supernatant from LPS-treated macrophages. Stimulation index decreased significantly in the presence of the supernatants of 72-hour LPS-treated macrophages from BALB/c and C57BL/6 mice. There was no significant difference between the inhibitory effect of the supernatants of BALB/c and C57BL/6 macrophages.

### Phagocytosis assay

**Fig 6:**
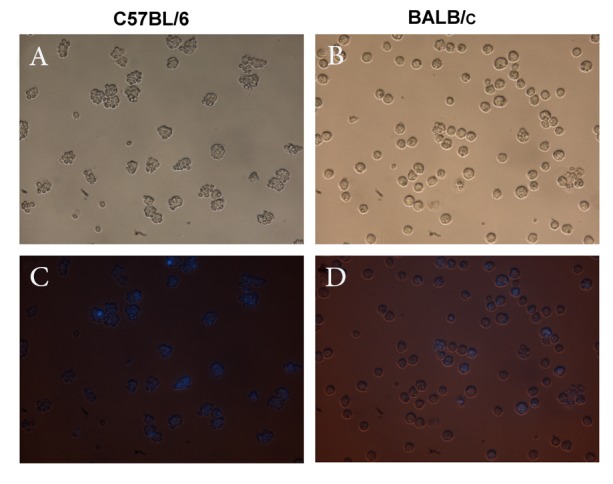
Phagocytosis of DAPI-stained Saccharomyces cerevisiae by C57BL/6 and BALB/c macrophages at 72 hours post-LPS treatment. A, B represent phase contrast and C, D represent florescent microscopy images of the macrophages after incubation for one hour with Saccharomyces cerevisiae (×400).

**Fig 7:**
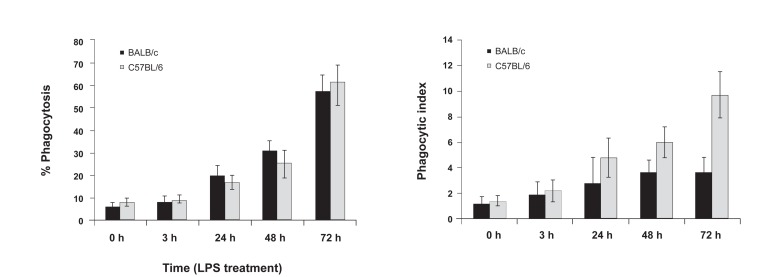
The mean numbers of macrophages that contain Saccharomyces cerevisiae per total macrophages at 0 (pre-treatment), and 3, 24, 48 and 72 hours post-LPS treatment (7 A). The meannumber of Saccharomyces cerevisiae ingested per phagocyte at 0 (pre-treatment), and 3, 24, 48, and 72 hours post-LPS treatment (7 B). The single star on top of the bars shows significant difference between BALB/c and C57BL/6 groups.

LPS-treated peritoneal macrophages from BALB/c, and C57BL/6 mice were incubated for 1hour with DAPI stained Saccharomyces cerevisiae at a 5: 1 ratio. After the incubation period,we analyzed the cells under a fluorescence microscope (Fig 6). In the different microscopic fields that were analyzed, we counted the number of cells that had internalized Saccharomyces cerevisiae and the number of internalized Saccharomyces cerevisiaeper cell. The mean numbers of cells that contained Saccharomyces cerevisiae per total cell were reported as percent of phagocytosis (Fig 7A). The number of Saccharomyces cerevisiae ingested per phagocytewas reported as the phagocytic index (Fig 7B). There was significant phagocytosis in both mice groups after 24 hours of LPS treatment. In addition, longer incubation with medium that containedLPS had a positive effect on phagocytosis. There was no significant difference between the percent of phagocytosis between BALB/c and C57BL/6 mice. According to the phagocytic index (Fig 7B), LPS treatment had a stimulatory effect on the number of internalized Saccharomyces cerevisiae. There was a significant difference between BALB/c and C57BL/6 mice according to the phagocytic index. As shown in figure 7, the mean number of internalized Saccharomyces cerevisiae after 72 hours of LPS treatment in C57BL/6 macrophages (9.7) was significantly higher than observed with BALB/c macrophages (3.6).

## Discussion

Numerous studies of macrophage function have shownvarious stimulators such macrophage responses to specific stimulatorssuch as LPS. These studies encompass the effects of LPS on genetic reprogramming and the expression of pro-inflammatory genes (11, 13), the signal transduction of LPS/TLR4 (6, 14, 15), inhibitors and stimulators of LPS function (7, 16-20), and macrophage migratory and inflammatory response to LPS (10, 21). In spite of that, all studies have done with the same cells called macrophages, and the same stimulus called LPS; we see discrepancies in results that make it difficult to use the same stimulus like LPS in a correct way for obtaining the favorite result. According to aliterature review, variationsin macrophage preparation and genetic background explain these discrepancies.

Ghosn et al. (9) have shown that the peritoneal cavity contains two different macrophage subsets, LPMs and SPMs that differ developmentally, physically, and functionally. They demonstrated that after thioglycolate stimulation, a population of blood monocytes infiltrate into the peritoneal cavity and mature as SPMs. SPMs express CD62L (L-selectin) and can migrate to other lymphoid organs as antigen presenting cells (APCs). Previous studies have shown that macrophages can be divided functionally to M-1/M-2 (5) according to their cytokine pattern and their effective role in the induction of Th1/Th2 in surrounding lymphocytes. In the current study, we have compared the effects of LPS on the function of thioglycolate-stimulated peritoneal macrophages in BALB/c and C57BL/6 mice as representatives of the Th2 and Th1 immune response, respectively. After separation of the peritoneal macrophages, their purity was confirmed by flowcytometry measurement of the CD11b marker. Our results showed thatthioglycolate stimulation induced CD14 expression in 93% of peritoneal macrophages. Fock et al. (21) demonstrated that only about 58% of peritoneal macrophages expressed CD14 without stimulation. Macrophages responded to LPS in the presence of CD14 and the TLR-4/MD-2 receptor complex.This result has shown that pre-separation thioglycolate stimulation may prepare macrophages to respond better to the next *in vitro* LPS stimulation.There was no difference between CD14 expression in BALB/c and C57BL/6 macrophages. A time course study of macrophage cytokine production after 1 µg/ ml LPS treatment showed that LPS could induce cytokine production of thioglycolate-stimulated macrophages. There were more TGF-β_1_ cytokines (1500 pg/ml) produced by BALB/c macrophages compared to those produced by C57BL/6 macrophages (400 pg/ml) at 72 hours post-LPS treatment.A larger quantity of IFN-λ cytokines (130 pg/ml) were produced by C57BL/6 macrophages compared to the quantity of IFN-λ cytokines (30 pg/ml) produced by BALB/c macrophages at 72 hours post-LPS treatment. These results confirmed the dominant M-2/M-1 responses of BALB/c and C57BL/6 macrophages. Our results indicated that during the first hours after LPS treatment the quantity of IL-17 and IL-10 produced by C57BL/6 macrophages was significantly higher than BALB/c macrophages; after 72 hours of LPS treatment both macrophages produced the same quantity of IL-17 and IL-10 cytokines. This result was the first comparative report on IL-17 and IL-10 production by LPS-stimulated peritoneal macrophages. The results have suggested that the different pattern of IL-17 and IL-10 cytokine production at the first 72 hours after LPS treatment in C57BL/6 and BALB/c macrophages were determinant in the formation of the M-1/M-2 microenvironment and induction of Th1/Th2 immune responses. As indicated in figure 3, IL-4 production by BALB/c macrophages wassignificantly higher than C57BL/6 macrophages at 72 hours after LPS treatment. The results demonstrated that during thefirst hours after LPS treatment, BALB/c macrophages produced more nitrite than C57BL/6 macrophages. After 72 hours, C57BL/6 macrophages produced significantly more nitrite compared to BALB/c macrophages. This result contrasted the previous reported results on the inhibitory effect of TGF-β_1_ production on iNOS activity and nitrite production (22, 23). This contrast has explained the bi-directional modulatory effect of TGF-β_1_ on macrophages. TGF-β_1_ induces pro-inflammatory responses in resting macrophages, but induces anti-inflammatory responses in active macrophages according to their cytokine milieu (24).

In addition, the higher amount of nitrite production at 3 and 24 hours post-LPS treatment in BALB/c macrophages contrasted previous M2 macrophage behavior after LPS treatment. This could be explained by previous reports, which have shown that macrophages differentially metabolize arginine through arginase or iNOS activation, depending on the inflammatory condition (4, 25, 26). This contrast is related to different inflammatory conditions surrounding the BALB/c macrophagesin the first 24 hours in our experiment, which was induced by thioglycolate stimulation (9).

Previous studies have shown that activation of IDO in macrophages hasan inhibitory effect on T-cell proliferation (27). Some stimulators, like macrophage colony-stimulating factor or a combination of CD40 ligand and IFN-λ signals (28), can induce IDO activity. Our results have shown that both BALB/c and C57BL/6 macrophages have IDO activity, with no difference between the amounts of kynurenine produced between them. In addition, LPS stimulation has no significant effect on IDO activity. In order to study the thioglycolate-stimulated macrophage effect on splenocyte proliferation, splenocytes of F1 hybrid BALB/c×C57BL/6 mice were cultured in the presence of LPS-stimulated macrophage supernatants. To eliminate the splenocyte response to foreign antigens in the supernatant of macrophage cultures and employing the same source of splenocyte, F1 BALB/c×C57BL/6 mice were used for splenocyte preparation (4). The results showed that macrophage supernatants significantly inhibited splenocyte proliferation at 72 hours post-LPS treatment. This result agreed with the increase in IDO activity at 72 hours post-LPS treatment in both BALB/c and C57BL/6 mice, and increased IL-10 and TGF-β_1_ production in C57BL/6 and BALB/c mice, respectively.Phagocytosis is a complex process in macrophages, whichare dependent on both internal and environmental characteristics. A phagocytosis assay can reflect the power of the pathogen to resist elimination and the ability of different macrophages and different macrophage stimulators to combat these pathogens (3, 16, 29). In this study, we have useda phagocytosis assay to compare different genetic background effects on phagocytosis. According to figure 7, we determined that LPS stimulated phagocytosis in both BALB/c and C57BL/6 macrophages. Data has shown no significant difference in the number of cells that phagocyte *Saccharomyces cerevisiae*, but C57BL/6 macrophages have greater phagocytic capacity. This result indicates that macrophages with different genetic backgrounds will respond to the same stimuli with different kinetics and in a different capacity. Variations in quality and quantity of phagocytic capacity, cytokine, and NO production at different times after the encounter to the same stimulus can explain the reason for different immune outcomes between BALB/c and C57BL/6 mice in response to infections.

## Conclusion

This comparative study shows that BALB/c and C57BL/6 macrophages show different immune response to LPS stimulation according to their Th2/ Th1 potential. Some discrepancies exist between BALB/c and C57BL/6 macrophages in terms of their Th2 and Th1 behavior in immunologic response to LPS. These discrepancies show that LPScan modulate immune responses in different ways, not only depending on genetic background, but also on the current inflammatory context of the immune system induced bythioglycolate stimulation.

This study represents the time course of immune response events of macrophages to LPS stimulation.The results enable us to select the best time for additional analyses in futureexperiments.
